# Neutrophil to C-reactive protein ratio: An estimating factor for intestinal ischemia before the surgery of incarcerated inguinal hernia

**DOI:** 10.14744/nci.2021.26878

**Published:** 2021-12-31

**Authors:** Kemal Eyvaz, Onur Ilkay Dincer, Murat Kazim Kazan, Aydın Dincer, Arif Aslaner, Aylin Acar, Tugrul Cakir

**Affiliations:** 1. Department of General Surgery, University of Health Sciences Antalya Training and Research Hospital, Antalya, Turkey; 2. Department of General Surgery, University of Health Sciences Umraniye Training and Research Hospital, Istanbul, Turkey

**Keywords:** Emergency surgery, estimating factor, incarcerated inguinal hernia, neutrophil to C-reactive protein ratio

## Abstract

**Objective:**

An inguinal hernia may transform to an incarcerated hernia, which would require emergency surgery with increased morbidity and mortality. This study aims to analyze whether it is possible to predict intestinal ischemia in incarcerated hernia using complete blood count parameters and serum C-reactive protein (CRP).

**Methods:**

Medical records of 129 patients were used to analyze whether there is a correlation between intestinal ischemia and laboratory parameters. Receiver operating characteristics analysis and Youden index were used to determine cutoff values, sensitivity, and specificity.

**Results:**

Female patients, those with a femoral type hernia, and patients with additional diseases were found to be more prone to bowel resection. CRP, lymphocyte to CRP ratio (LCR), and neutrophil to CRP ratio (NCR) parameters were significant (AUC=0.914, p<0.001; AUC=0.901, p<0.001; and AUC=0.908, p<0.001, respectively). A value <0.45 for NCR has a sensitivity of 93.3% and specificity of 87.8%; a value >19 in CRP has a 90% sensitivity and 88.9% specificity.

**Conclusion:**

Decreased pre-operative NCR and LCR, and increased CRP levels can be used as a predictor for estimating intestinal ischemia.

**I**nguinal hernia is a frequently observed diagnosis, especially among the elderly, and due to its risk of incarceration, surgery is the unique treatment option [1]. Inguinal hernia repair may require much more health-care resources when it is incarcerated; and it has a high lifetime risk of incarceration; 27% for men and 3% for women [2]. Inguinal, femoral, incisional, umbilical, epigastric, and parastomal herniae are other type of abdominal wall hernias, but femoral hernias tend to be more complicated [3]. Incarceration is defined as trapping of the contents within the hernia sac and when reducing them back into the abdomen is not possible. If ischemia and necrosis of the contents develop, this is called strangulation. The risk of incarceration and strangulation of inguinal hernia varies between 0.3% and 3%/yea [4, 5]. Factors such as older age, femoral type of groin hernia, and recurrent hernia are associated with incarceration [6–8]. Resection of the strangulated segment is associated with increased post-operative morbidity [9–11]. In the literature, there are plenty of studies which try to find out the exact levels of certain blood parameters that indicate the severity of inflammation or ischemia [12–14].

In this retrospective study, we aimed to evaluate our surgical results of emergency inguinal hernia operations and the relationship of different laboratory parameters to determine the strangulation of intestinal segments before surgery.

## Materials and Methods

Medical records of 129 patients, who were operated in a tertiary hospital with a diagnosis of incarcerated inguinal hernia between September 2015 and December 2020, were collected with the help of the electronic records of the hospital. Indication of operation was decided by a general surgeon in patients who had signs of intestinal obstruction such as vomiting and irreducible swelling in the groin area and who had positive findings on plain abdominal X-ray. Patients who were pregnant, had cirrhosis, had any active systemic infection or those with any comorbid diseases that may affect inflammation, a hematological disease, those that were under the age of 18, and those who did not provide written informed consent were excluded from the study.

Patients were categorized into two groups; one consisted of patients who underwent intestinal resection (Group R) and the other one consisted of patients without any resection (Group NR). Demographics records such as patient’s age, gender, ASA score, comorbid disease presentation (diabetes mellitus, chronic pulmonary diseases, coronary disease, and hypertension), type of anesthesia, and type of hernia (inguinal and femoral) were recorded. Pre-operative complete blood count parameters: White blood cell (WBC), neutrophil, lymphocyte, and serum C-reactive protein (CRP) levels recorded for analysis. Neutrophil to lymphocyte (NLR), lymphocyte to CRP (LCR), and neutrophil to CRP (NCR) ratios were calculated to evaluate whether there is a correlation with strangulation. All the data collected are documented through Office 365 Excel worksheet version 16.41 (Microsoft Corp., Redmond, WA). The study was approved by the University of Health Sciences Antalya Training and Research Hospital Ethical Committee with an 2021/033 number at March 4, 2021.

**Highlight key points**

• Intestinal ischemia of incarcerated inguinal hernia can be predicted with simple blood tests.
• CRP levels increase in patients with intestinal ischemia.
• Neutrophil to CRP level (NCR) <0.45 could be used as an estimating factor for intestinal ischemia before surgery.
• The NCR could become a valuable parameter in estimating the necessity of bowel resection in the near future.
• To the best of our knowledge, there is no report on the use of NCR in the prediction of intestinal ischemia.

### Statistical Analysis

All statistical analyses were carried out using JMP version 15.1 (SAS Institute Inc., Cary, NC, 1989–2019). Normality analysis of the data was tested using Shapiro–Wilk test. As the continuous variables were normally distributed; descriptive statistics are shown as median±interquartile range (IQR). Categorical variables are displayed using numbers (n) and percentages (%). Chi-square test or Fisher’s exact test was performed for sex, type of hernia, type of anesthesia, and mortality rate comparisons between groups. Independent t-test was performed in the comparison of age, length of hospital stay (days), WBC, neutrophil count, lymphocyte count, CRP, NLR, NCR, and LCR values of the groups. Receiver operating characteristics (ROCs) analysis performed to determine the cutoff value of the parameters between groups. The area under curve (AUC) and 95% confidence intervals calculated. In general, the value of AUC was accepted as follows; 0.9–1: excellent; 0.8–9: good; 0.7–0.8: fair; 0.6–0.7: poor; and 0.5–0.6: fail. The Youden index is used for determining the best cutoff points in the ROC analysis. P<0.05 was accepted as statistically significant.

## Results

In the scope of our study, there were 139 patients operated with the diagnosis of incarcerated inguinal hernia. The patients who had additional inflammatory comorbidities and missing laboratory test results were excluded from the study. The results of a total of 129 patients were included in our study. Median age was 65 years, with a value of IQR of 28.5. There were 38 (29%) female and 91 (71%) male patients. Ninety-six (74%) of the patients were operated with a diagnosis of incarcerated inguinal hernia, while 33 (26%) had a femoral hernia. Sixty-nine of the patients (53%) had concomitant diseases such as diabetes mellitus, hypertension, and coronary artery disease. Median hospital stay was 2 days (3; IQR). Ninety-nine (76, 7%) patients were in Group R; while 30 (23, 3%) patients were in Group NR. Length of hospital stay, concomitant disease, and mortality rates were statistically significant between Group R and Group NR (p=0.004, p<0.001, and p=0.012; respectively). Other demographic data of patients are listed in Table 1.

**Table 1.** Demographic value of patients

	All (n=129) %	Resection (n=30) %	No resection (n=99) %	p
Gender				
Female	29	47	24	**0.018**
Male	71	53	76
Age, years*	65 (50–79)	79 (66–84)	63 (47–75)	**<0.001**
Type of hernia				
Inguinal	74	60	79	**0.038**
Femoral	26	40	21	
Concomitant disease				
+	53	80	45	**<0.001**
–	47	20	55	
ASA score				
1 and 2	77	43	82	**<0.001**
3 and 4	23	57	18	
Anesthesia type				
General	45	90	31	**<0.001**
Regional	55	10	69	
Mortality				
+	3	10	1	**0.012**
–	97	90	99	
Length of hospital stay, days	2 (1–4)	5 (2–8)	2 (1–3)	**0.004**

*: Data are present as median and quartiles; ASA: American Society of Anesthesiologists.

We performed univariate analysis of the complete blood count parameters and serum CRP levels and calculated some ratios between these results. CRP, LCR, and NCR ratios are statistically significant between Group R and Group NR (p<0.001, p<0.001, and p=0.03; respectively). Other laboratory parameters are listed in Table 2.

**Table 2.** Comparison of hematological and biochemical parameters

	Group R (Median–quartiles)	Group NR (Median–quartiles)	p
WBC (×10^3^/mm^3^)	11.7 (10.4–14.8)	9.3 (8–11.5)	<0.001
Neutrophil (×10^3^/mm^3^)	10 (8.1–12.9)	6.7 (5.2–9.7)	<0.001
Lymphocyte (×10^3^/mm^3^)	1.1 (0.7–1.6)	1.5 (1–2.2)	0.01
Platelet (×10^3^/mm^3^)	245 (203–303)	242 (204–284)	0.95
CRP (mg/dl)	74.5 (29.3–149.7)	5 (2–11)	<0.001
NLR (×10^3^)	9.5 (5.5–14.5)	4.8 (2.3–8.3)	0.03
LCR (×10^3^)	0.014 (0.005–0.05)	0.32 (0.12–0.82)	<0.001
NCR (×10^3^)	0.11 (0.07–0.25)	1.42 (0.71–3)	<0.001

WBC: White blood cell; CRP: C-reactive protein; NLR: Neutrophil to lymphocyte ratio; LCR: Lymphocyte to C-reactive protein ratio; NCR: Neutrophil to C-reactive protein ratio.

ROC analysis was performed for complete blood count parameters, CRP levels, and ratios between them to determine the cutoff values between groups. Results revealed that WBC, CRP, LCR, and NCR parameters were statistically significant (AUC=0.728, p<0.001; AUC=0.914, p<0.001; AUC=0.901, p<0.001; and AUC=0.908, p<0.001; respectively, demonstrated in Table 3).

**Table 3.** Receiver operating characteristics analysis of parameters for the prediction intestinal resection

	Value	AUC (95% CI)	Sensitivity (%)	Specificity (%)	p
WBC (×10^3^/mm^3^)	10.3	0.728 (0.63–0.827)	83.3	65.7	<0.001
Neutrophil (×10^3^/mm^3^)	8.5	0.715 (0.611–0.818)	76.6	70.7	<0.001
CRP (mg/dl)	19	0.914 (0.851–0.977)	90	88.9	<0.001
NLR (×10^3^)	8	0.713 (0.606–0.82)	70	72.7	<0.001
LCR (×10^3^)	0.1	0.901 (0.84–0.963)	90	78.8	<0.001
NCR (×10^3^)	0.45	0.908 (0.836–0.98)	93.3	87.88	<0.001

AUC: Area under curve; CI: Confidence interval; WBC: White blood cell; CRP: C-reactive protein; NLR: Neutrophil to lymphocyte ratio; LCR: Lymphocyte to C-reactive protein ratio; NCR: Neutrophil to C-reactive protein ratio.

**Figure 1.**
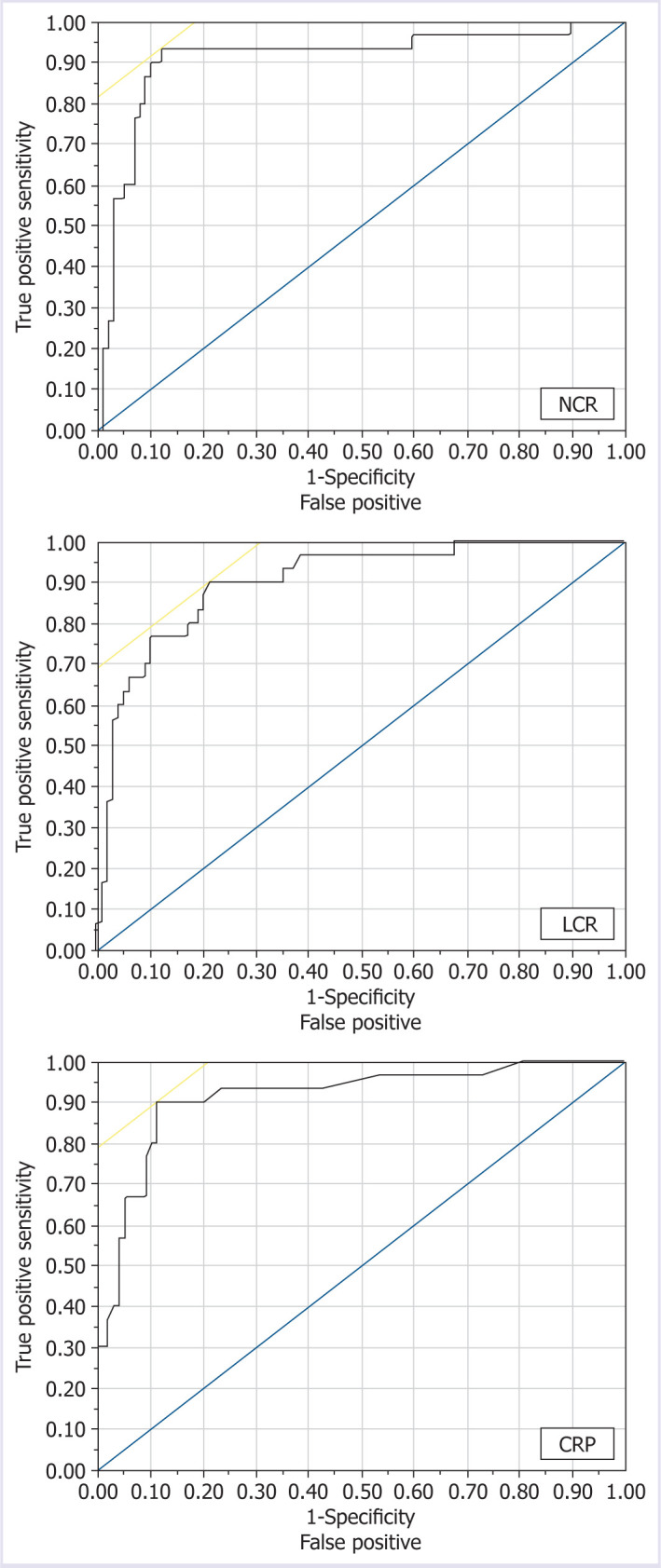
Receiver operating characteristic curve analysis of for NCR, LCR, and CRP. NCR: Neutrophil to C-reactive protein; LCR: Lymphocyte to C-reactive protein; CRP: C-reactive protein.

The cutoff value for NCR is 0.45, and for CRP, it is 19 to predict the intestinal resection before surgery. A value <0.45 for NCR has a sensitivity of 93.3% and a specificity of 87.8%; and a CRP value over 19 has 90% sensitivity and 88.9% specificity. The ROC analysis and results are shown in Figure 1.

## Discussion

Postponed elective groin hernias may lead to the incarceration of intra-abdominal organs over time, requiring emergency surgery, which may result in increased morbidity and mortality. Patients who underwent emergency hernia repair with bowel resection due to the necrosis of the intestinal segments are more likely to experience longer hospitalizations and higher post-operative complications ranging from 6% to 43%; and higher mortality rates ranging from 1% to 7% [9, 10, 15]. We find our overall mortality rate to be 3%, while this ratio is 10% in Group R, similar to the literature. A meta-analysis performed by Chen et al. [16] including seven studies and a total of 762 patients; female gender, age (>65 years), femoral hernia, intestinal obstruction, total time of incarceration (hours), leukocyte, and neutrophil count were found to be associated with intestinal resection. In our study, female gender, femoral type hernia, and patients with additional diseases were found to be more likely to require bowel resection. Median age was 70 years (66–84) in Group R. An important point is which incision and technique will be used for the repair. If the surgeon thinks that bowel resection may be required before starting surgery, laparotomy or laparoscopy may be the first option. There are some studies that try to predict intestinal ischemia, necrosis, or gastrointestinal cancers. Complete blood count is easy to perform and parameters such as WBC, lymphocyte, neutrophil, thrombocyte counts, and various ratios of these parameters are studied [17–19]. NLR is the most studied parameter in acute infectious situations such as acute pancreatitis or mesenteric ischemia [20, 21]. In a study carried out with 323 patients; increased NLR accompanied by bowel obstruction was shown to potentially result in bowel resection [22]. We found that an NLR value >8 may result in bowel resection, with 70% sensitivity and 72.7% specificity (p<0.001). NLR can be used as a marker for the prediction of intestinal ischemia but the sensitivity and specificity of NLR are not quite high, therefore, additional markers are needed to be studied.

CRP is a widely used inflammatory marker; the level of CRP may increase up to 1000 times based on the severity of inflammation and response of the body to the inflammation [23]. Korkut et al. [24] mentioned that CRP levels can be a marker in discriminating complicated appendicitis and simple appendicitis. The cutoff value of CRP was found to be 125, with 64% sensitivity and 91% specificity, in patients operated with a diagnosis of incarcerated inguinal hernia in a study carried out by Yildirim et al. [25] In our data, there is a statistically significant difference between the Groups R and NR in terms of CRP levels. The cutoff value of CRP was calculated to be 19 with 86.6% sensitivity and 91% specificity. CRP can be used to evaluate the necessity of bowel resection before the operation. In the literature, there are few studies that mention the value of CRP in determining the need for resection.

LCR is another marker which was used to evaluate the surgical and oncological outcomes in certain gastrointestinal cancers [26, 27]. The LCR level is also used to evaluate the complexity of acute appendicitis and intestinal necrosis in incarcerated inguinal hernia [21, 28]. In our study, we found that LCR levels <0.1 are associated with intestinal resection with a 90% sensitivity and 78.8% specificity (p<0.001).

The NCR could become a valuable parameter in estimating the necessity of bowel resection in the near future. In acute inflammation, an increase in WBC count also lead to an increase in the neutrophil count. A greater increase in the CRP value is observed, when compared to the neutrophil count, in terms of proportionality to normal values; meaning that the increase in CRP is much higher with respect to its baseline level. Therefore, the NCR could be an early sign of inflammation. The increase in NLR gives us valuable information but the NCR is more valuable than NLR. To the best of our knowledge, there is no report on the use of NCR in the prediction of intestinal ischemia. The cutoff value of NCR was calculated as 0.45. Patients who have an NCR value <0.45 may be more prone to resection with 93.3% sensitivity and 87.8% specificity (p<0.001).

### Limitations

Since this is a retrospectively designed study, we have some limitations. Data were collected from the database of our hospital and there may be some missing values due to coding errors. Even though the study was conducted in a tertiary hospital, it is a single-centered study and our results need to be supported in multicentric prospective trials.

However, to the best of our knowledge, the present study is the first study showing that low pre-operative NCR levels could be used as an indicator to predict necrosis of the intestinal segment in patients operated with incarcerated inguinal hernia.

### Conclusion

Incarcerated groin hernia is a vital surgical problem which may lead to increased mortality. A neutrophil to CRP ratio <0.45 can be used as an indicator to predict intestinal ischemia before surgery.
